# Editorial: Multimodal brain data integration and computational modeling

**DOI:** 10.3389/fninf.2026.1838147

**Published:** 2026-05-11

**Authors:** Jony Sheynin, Maryam Naseri, Alejandro Luis Callara

**Affiliations:** 1 Department of Psychiatry and Behavioral Science, Texas A&M University Health Science Center, Houston, TX, United States; 2 Department of Radiology and Biomedical Imaging, Yale School of Medicine, New Haven, CT, United States; 3 Department of Information Engineering and Research Center “E. Piaggio”, University of Pisa, Pisa, Italy

**Keywords:** deep learning, artificial intelligence, neuroimaging, machine learning (ML), microscopy, multimodal data fusion, Magnetic Resonance Imaging (MRI), precision medicine

Brain function cannot be fully characterized by any single modality. Instead, each imaging technique offers a distinct balance of temporal and spatial resolution (Zhang et al., 2020). EEG and MEG capture millisecond-level neural dynamics, whereas fMRI and PET offer greater spatial specificity and sensitivity to hemodynamic or molecular processes. This fundamental trade-off has long shaped how researchers investigate fast neural dynamics vs. large-scale network organization (Horwitz and Poeppel, 2002). Over the last two decades, multimodal neuroimaging has emerged as a powerful strategy to bridge these limitations. Simultaneous acquisitions—such as EEG/fMRI or MEG/fMRI—together with computational fusion frameworks, including Bayesian and other generative or model-based approaches, now enable more comprehensive and biologically-grounded characterizations of brain activity (He and Liu, 2008; Trujillo-Barreto et al., 2023).

From a structural point of view, additional integration is possible, from structure-function relationships to across-scale integration: Microstructural organization provides a bridge to mesoscale and macroscale organization and can constrain functional dynamics in integrative models (Amunts and Zilles, 2015; Honey et al., 2009).

Further, multimodal brain science increasingly extends beyond imaging to genetic, behavioral, and clinical variables, pushing the field from pairwise associations toward multivariate and machine-learning methods tailored to high-dimensional, heterogeneous datasets (Liu and Calhoun, 2014; Bogdan et al., 2017). Importantly, such incorporation of individual biomedical data from various domains informs clinical decision-making and supports the development of patient-specific interventions (Pham et al., 2024).

This Research Topic, *Multimodal brain data integration and computational modeling*, was conceived to: (i) Explore strategies for the effective fusion of heterogeneous neural data; (ii) Present computational and machine-learning approaches for modeling complex brain processes; and (iii) Demonstrate applications in basic and clinical neuroscience. The included articles are organized thematically below. Across the Research Topic, a recurring message is that integration is not only across modalities, but also across levels of description—from genes and microstructure to systems-level brain function.

A first set of contributions emphasizes multilevel integration, combining molecular, cellular, and microstructural measures with systems-level functional organization. Nazac et al. review recent advances in super-resolution microscopy and deep learning-based segmentation for neuronal microstructure, highlighting how modern computational pipelines enable quantitative characterization of features such as dendrites and spine morphology from high-resolution imaging modalities (e.g., SIM, STED, STORM, and MINFLUX). Piszczek et al. propose an integrative workflow that combines evolutionary genetic data with searchable, psychologically organized fMRI resources, using a genetic algorithm for biclustering (GABi) to identify gene sets under selection whose expression profiles relate to functional networks. Together, these studies underscore that progress in multimodal neuroscience depends not only on acquiring diverse data, but also on building analysis frameworks that connect biological mechanisms to interpretable, system-level models across scales.

A second set of contributions advances multimodal integration by learning cross-modal mappings that mitigate practical constraints such as data scarcity, cost, and radiation exposure. Revathy and Karthiga introduce AutismSynthGen, a privacy-preserving framework for autism spectrum disorder (ASD) prediction that synthesizes multimodal ASD data—including structural MRI, EEG, behavioral measures, and severity scores—using a transformer-based generative model with differential privacy coupled with an adaptive mixture-of-experts ensemble to improve predictive performance. Ferrante et al. propose a deep-learning approach to synthesize TSPO-PET images from structural MRI, aiming to approximate PET-derived neuroinflammation-related information from a more widely available modality; the generated images reproduce key spatial signal patterns and contrast properties observed in real TSPO-PET scans. Together, these studies illustrate how cross-modal learning and synthesis can make multimodal assessments more feasible and scalable, while preserving clinically relevant information when direct acquisition is limited.

A third theme focuses on robust inference and prediction, addressing noise, dimensionality, and heterogeneity in electrophysiology and connectivity. Patel et al. present a hybrid framework (DWT-CNN-BiGRU) for the classification of EEG signals from alcoholic and control subjects, combining wavelet-based denoising with convolutional and recurrent components to capture spatial and temporal structure and improve performance in a clinically relevant task. This contribution highlights how careful preprocessing and principled statistical learning can strengthen generalizable prediction from noisy, high-dimensional brain signals—an essential requirement for scalable multimodal modeling.

Collectively, the contributions in this Research Topic reflect a converging direction for the field: Multimodal integration increasingly requires methods that bridge biological scales, learn informative cross-modal representations under real-world constraints, and deliver robust, generalizable inference. Continued progress will benefit from shared benchmarks, transparent validation practices, and models that balance predictive performance with interpretability. This, in turn, would support reproducible multimodal neuroscience and facilitate the development of clinical applications and precision approaches, to improve prediction of outcomes and treatment response.
